# Wide identification of chemical constituents in fermented licorice and explore its efficacy of anti-neurodegeneration by combining quasi-targeted metabolomics and in-depth bioinformatics

**DOI:** 10.3389/fnins.2023.1156037

**Published:** 2023-05-18

**Authors:** Xiaotong Wang, Ying Liu, Nianxin Kang, Guojie Xu

**Affiliations:** School of Life Sciences, Beijing University of Chinese Medicine, Beijing, China

**Keywords:** licorice, natural medicinal products, probiotics, quasi-targeted metabolomics, bioinformatics

## Abstract

Licorice (Gan-Cao in Chinese) is one of the most famous herbal medicines around the world. The fermentation of probiotics and herbs can change the chemical constituents and significantly improve the efficacy. However, it is still unknown whether licorice fermented with probiotics would produce beneficial therapeutic effects. This study aimed to comprehensively analyze the chemical constituents in fermented licorice via quasi-targeted metabolomics, predict the potential efficacy of fermentation products via diverse bioinformatic methods, and further verify the efficacy of fermentation products through *in vitro* and *in vivo* experiments. As a result, 1,435 compounds were identified totally. Among them, 424 natural medicinal products were classified with potentially important bioactivities, including 11 anthocyanins, 10 chalcones and dihydrochalcones, 25 flavanones, 45 flavones and flavonols, 117 flavonoids, 34 isoflavonoids, 21 phenols and its derivatives, 20 phenylpropanoids and polyketides, 96 terpenoids and 25 coumarins and derivatives. Interestingly, bioinformatic prediction showed that the targets of some important compounds were related to neurodegeneration, oxidoreductase activity and response to stress. *In vitro* and *in vivo* tests further verified that fermented licorice had excellent effects of DPPH clearance, anti-oxidation, anti-neurodegeneration, and anti-stress. Thus, this study would provide a reference method for related research and the development of fermented licorice-related products.

## 1. Introduction

Licorice (Gan-Cao in Chinese), mainly derived from the root and rhizome of *Glycyrrhiza* species, is one of the most famous herbal medicines. Licorice has been widely used to treat various chronic diseases in Asia for thousands of years. And now, licorice is also used as an additive in cosmetics, food, and animal husbandry. With the increasing demand of licorice, *G. uralensis* is widely cultivated in China, with an annual production of over 5,000 tons (Shibata, 2000; Hosseinzadeh and Nassiri-Asl, 2015; Pastorino et al., 2018; Jalali et al., 2021).

Licorice contains a wide variety of chemical compounds, mainly including triterpenoids and flavonoids, which has a wide range of biological activities, including anti-inflammation, anti-virus, anti-tumor (Asl and Hosseinzadeh, 2008; Abraham and Florentine, 2021; Heidari et al., 2021; Sharifi-Rad et al., 2021; Wahab et al., 2021). Some studies also reported that licorice had the efficacy of preventing severe acute respiratory syndrome (SARS) (Fiore et al., 2008) and coronavirus disease (COVID-19) (Boozari and Hosseinzadeh, 2021; Brendler et al., 2021; Jalali et al., 2021; Liana and Phanumartwiwath, 2022).

Probiotics, defined as good bacteria in the human body, is one of the hotspots in current study. It had shown that probiotics had a wide range of biological activities, including maintaining the structural balance of intestinal flora, improving immunity and inhibiting inflammation (Sarao and Arora, 2017; Suez et al., 2019; Wieërs et al., 2019; Yu et al., 2020; Żółkiewicz et al., 2020). The latest research showed that the co-fermentation of probiotics and herbs could change the composition of ingredients and significantly improve the efficacy (Xiao et al., 2017; Han and Kim, 2020). However, it is still unknown whether licorice co-fermented with probiotics would produce beneficial therapeutic effects.

Quasi-targeted metabolomics is a novel metabolomic detection technology that combines the advantages of high throughput of non-targeted metabolomics with the advantages of high accuracy and sensitivity of targeted metabolomics, which is based on the SCIEX QTRAP® 6500+ mass spectrometer with triple quadrupole - linear ion trap composite and uses Multiple Reaction Monitoring (MRM) to accurately determine and quantify large amounts of metabolites in biological samples (Cao et al., 2022; Wang et al., 2022).

This study aimed to comprehensively analyze the chemical constituents in fermented licorice via quasi-targeted metabolomics, predict the potential functions of fermentation products via diverse bioinformatic methods, and further verify the therapeutic function of fermentation products through *in vitro* and *in vivo* experiments, so as to provide an important basis for the development of fermented licorice-related products.

## 2. Materials and methods

### 2.1. Licorice fermentation process

10 mg/mL licorice extract was fermented with 50 mg/mL probiotic complex (including *Lactobacillus plantarum* LP-115, *Streptococcus thermophilus* ST-21, *Lactobacillus casei* LC-11, *Bifidobacterium breve* BB-03, *Bifidobacterium infantis* BI-26, *Bifidobacterium lactis* BI-04, *Lactobacillus rhamnosus* GG, *Lactobacillus rhamnosus* HN001, Lactobacillus gratus LG-36, *Lactobacillus reuteri* 1E1, *Lactobacillus rhamnosus* LR-32, *Lactobacillus paracasei* LPC-37, *Bifidobacterium longum* BL-05, *Lactobacillus bulgaricus* LB-87, *Bifidobacterium lactis* HN019) in anaerobic medium at 37°C for 12 h.

### 2.2. Sample preparation

1 mL sample was lyophilized and suspended with 100 μL 80% methanol. The sample was incubated on ice for 5 min, centrifugated at 15,000 g, 4°C for 15 min. Supernatant of sample was diluted to final concentration of 53% methanol. The sample was subsequently centrifuged at 15,000 g at 4°C for 15 min and the supernatant was used for the LC–MS/MS analysis. An equal volume sample was taken from each experimental sample and mixed as a QC sample, and the blank sample was replaced by a 53% methanol aqueous solution (Want et al., 2006; Barri and Dragsted, 2013).

### 2.3. HPLC–MS/MS analysis

Xselect HSS T3 (2.5 μm, 2.1 × 150 mm) was kept at a flow rate of 0.4 mL/min for both the positive and negative polarity mode. Eluent A was 0.1% Formic acid-water and eluent B was 0.1% Formic acid-acetonitrile. The solvent gradient was set as follows: 2% B, 2 min; 2–100% B, 15.0 min; 100% B, 17.0 min; 100–2% B, 17.1 min; 2% B (Luo et al., 2015). LC–MS/MS analyses were performed using an ExionLC™ AD system (SCIEX) coupled with aQTRAP® 6500+ mass spectrometer (SCIEX). Positive polarity mode was set as follows: Curtain Gas of 35 psi, Collision Gas of Medium, IonSpray Voltage of 5,500 V, Temperature of 550°C, Ion Source Gas of 1:60, Ion Source Gas of 2:600 Negative polarity mode was set as follows: Curtain Gas of 35 psi, Collision Gas of Medium, IonSpray Voltage of −4,500 V, Temperature of 550°C, Ion Source Gas of 1:60, Ion Source Gas of 2:60 (Want et al., 2010; Dunn et al., 2011).

### 2.4. Chemical compound identification and quantification

MRM (Multiple Reaction Monitoring) were used to detect the signals of compounds based on in-house database. The Q1, Q3, RT (retention time), DP (declustering potential) and CE (collision energy) were used for compound identification. The Q3 were used for quantification (Wen et al., 2017).

### 2.5. Data analysis

Metabolites were annotated using the KEGG database,^1^ HMDB database^2^ and Lipidmaps database.^3^ BATMAN^4^ (Liu et al., 2016) and ToppGene^5^ (Chen et al., 2007, 2009a,b) were used for annotation of targets.

### 2.6. *In vitro* and *in vivo* test

DPPH clearance test was performed as follows: 200 μL of the sample was mixed with 200 μL DPPH (0.04 mg/mL) solution at room temperature for 30 min, and centrifuged at 5,000 r/min for 10 min. The supernatant was taken to measure the absorbance value at 517 nm, vitamin C was used as a positive control. The formula for calculating the DPPH clearance is: 1−(*A*1−*A*2)/*A*0*100%, where *A*0 is the absorbance value of mixed solution containing 400 μL absolute ethanol and 400 μL DPPH at 517 nm; A1 is the absorbance value of mixed solution containing 800 μL sample and 800 μL DPPH at 517 nm; A2 is the absorbance value of mixed solution containing 800 μL sample and 800 μL absolute ethanol at 517 nm.

The reducing ability test was performed as follows: 100 μL sample was mixed with 250 μL of phosphoric acid buffer of 0.2 mol/L (pH = 6.6) and then 250 μL of 1% potassium ferricyanide at 50°C for 20 min, 250 μL of 10% trichloroacetic acid was added to terminate the reaction. The sample was subsequently centrifugated at 5,000 r/min for 10 min. 500 μL of supernatant was collected and mixed with 500 μL of distilled water and 100 μL FeCl3, and allowed to stand for 10 min. The absorbance value was detected at 700 nm. Vitamin C as a positive control; The relative reduction capacity calculation formula is: absorbance of sample at 700 nm/absorbance of vitamin C at 700 nm * 100%.

Galactose-induced neurodegenerative *Caenorhabditis elegans* model was used to study the efficacy of fermented licorice (Cui et al., 2006; Caldwell et al., 2020). The control group was treated with 400 mM galactose, the other group was treated with 400 mM galactose containing different concentrations of fermented licorice, the number of swings of each *C. elegans* in 20 s was recorded. The heat stress capacity of *C. elegans* was detected as follows: *C. elegans* were treated with heat stress at 35°C, the number of survival *C. elegans* was counted every 2 h and further calculated.

## 3. Results and discussion

### 3.1. Comprehensive identification of chemical constituents of fermented licorice based on quasi-targeted metabolomics

**Figure 1:**
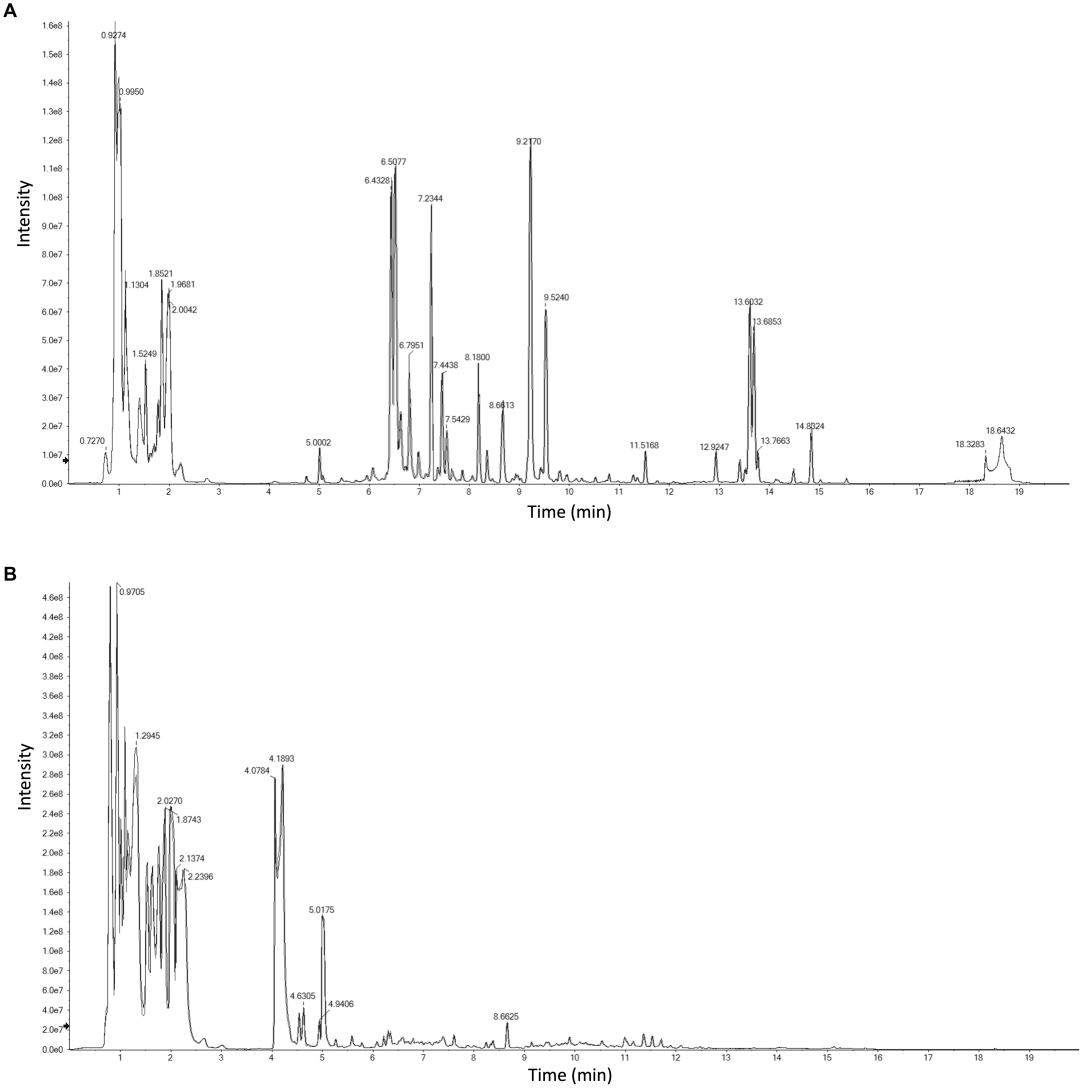
Total ion chromatorgraphy of mixed quality control sample of fermented licorice in positive **(A)** and negative **(B)** polarity mode.

The chemical ingredients of licorice are very complex, containing thousands of natural products, and similarly, there are hundreds of metabolites of probiotics. Current technologies are difficult to analyze such a large number of metabolites at one time. In order to comprehensively analyze the chemical constituents of probiotic fermented licorice, we used a triple quadrupole-linear ion trap complex SCIEX QTRAP® 6500+ mass spectrometer combined with multiple reaction monitoring mode (MRM) to accurately analyze the metabolites in probiotic fermented licorice. The results showed that the chromatographic peaks had good shape regardless of the positive ion or negative ion mode, and the quality control evaluation showed a good correlation, with an R2 value of 0.992 (close to 1), indicating that the conditions of liquid phase and mass spectrometry were stable and reliable (Figure 1). Qualitative identification results showed that we had identified 1,435 compounds in total, including compounds in licorice and many possible metabolites of probiotics (Supplementary Dataset S1).

Liquid chromatography coupled with mass spectrometry (LC/MS) was commonly used to analyze the chemical constituents of licorice (Montoro et al., 2011, Xu et al., 2013, Cheng et al., 2021, Shang et al., 2022a,b). Compared with previous studies, we firstly established the method to accurately identify the most chemicals in licorice-related researches via quasi-targeted metabolomics, which provided a reference method for related research.

### 3.2. Annotation of identified compounds

In order to understand the functional properties and classification of different compounds, we annotated the pathways and classifications of the identified compounds by using databases including Human Metabolome Database (HMDB), Kyoto Encyclopedia of Genes and Genomes (KEGG) and LIPID MAPS.

**Figure 2:**
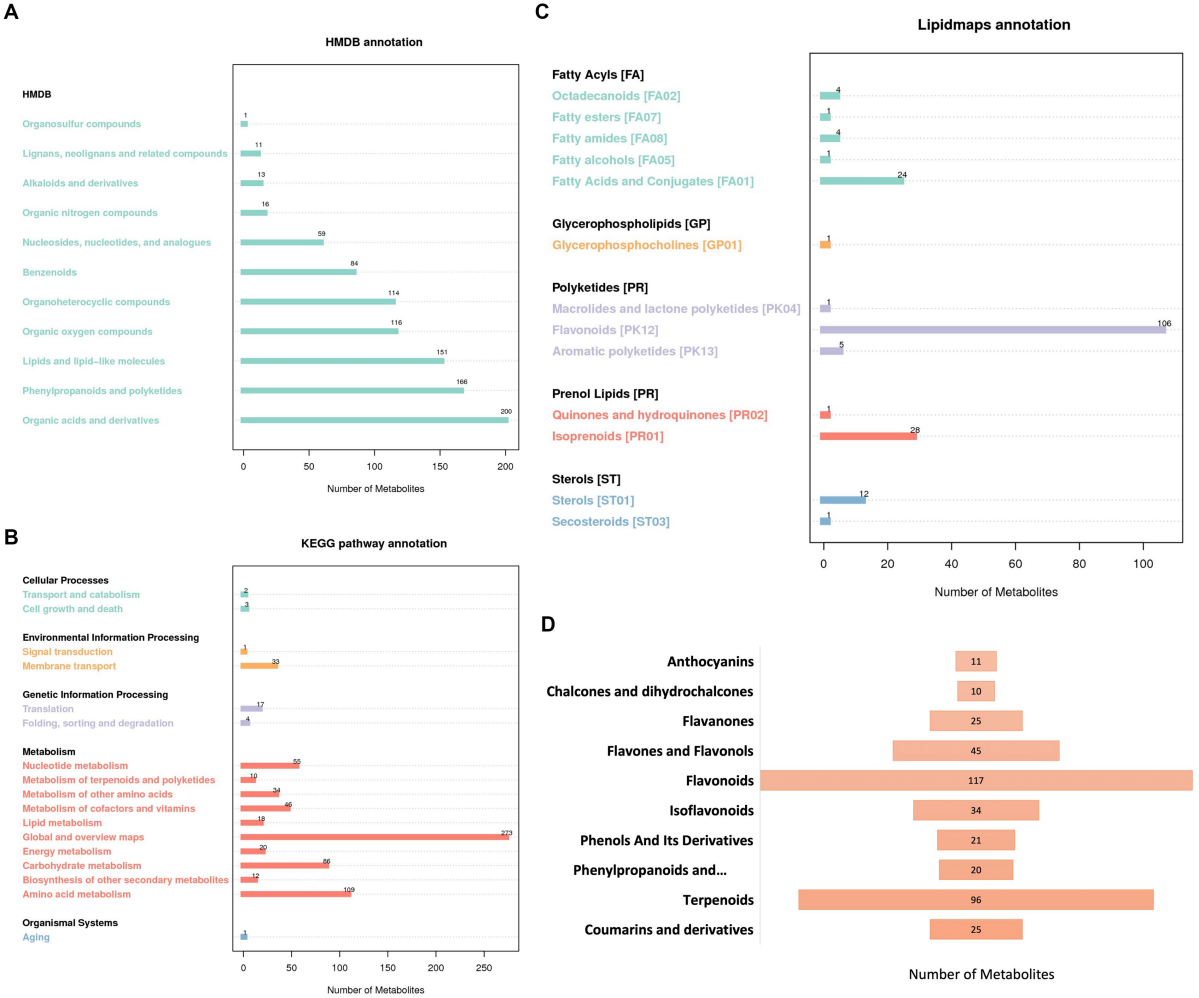
Annotation of 1,435 identified chemicals. **(A)** HMDB annotation. **(B)** KEGG pathway annotation. **(C)** Lipidmaps annotation. **(D)** Classification of 424 natural medicinal products.

HMDB annotation results showed that 1 compound belonged to organosulfur compounds, 11 compounds belonged to Lignans, neolignans and related compounds, 13 compounds belonged to alkaloids and derivatives, 16 compounds belonged to organic nitrogen compounds, 59 compounds belonged to nucleosides, nucleotides, and analogs, 84 compounds belonged to benzenoids, 114 compounds belonged to organoheterocyclic compounds, 116 compounds belonged to organic oxygen compounds, 151 compounds belonged to lipids and lipid-like molecules, 166 compounds belonged to phenylpropanoids and polyketides, and 200 compounds belonged to organic acids and derivatives (Figure 2A).

KEGG annotation results showed that 5 compounds were involved in cellular processes pathway, 34 compounds were involved in environmental information processing pathway, 21 compounds were involved in genetic information processing pathway, 663 compounds were involved in metabolism pathway, and 1 compound was involved in organic systems pathway (Figure 2B).

The LIPID MAPS annotation results showed that 34 compounds belonged to fatty acyls (FA), 1 compound belonged to glycerophospholipids (GP), 112 compounds belonged to polyketides (PK), 29 compounds belonged to prenol lipids (PR), and 13 compounds belonged to sterols (ST) (Figure 2C).

In general, this study not only identified the common natural products in licorice, but also found a lot of novel compounds produced by fermentation, which laid the foundation for the discovery of new active ingredients.

And more importantly, further sorting and classifying the compounds, we identified a total of 424 natural medicinal products with potentially important bioactivities, including 11 anthocyanins, 10 chalcones and dihydrochalcones, 25 flavanones, 45 flavones and flavonols, 117 flavonoids, 34 isoflavonoids, 21 phenols and its derivatives, 20 phenylpropanoids and polyketides, 96 terpenoids and 25 coumarins and derivatives (Figure 2D). These results demonstrated that the quasi-targeted metabolomics developed in this research was very suitable for the analysis of natural medicinal products, which could provide important support for the research on the new efficacy of fermented licorice and the development of new products.

### 3.3. Effect of fermentation on licorice chemical constituents

**Figure 3:**
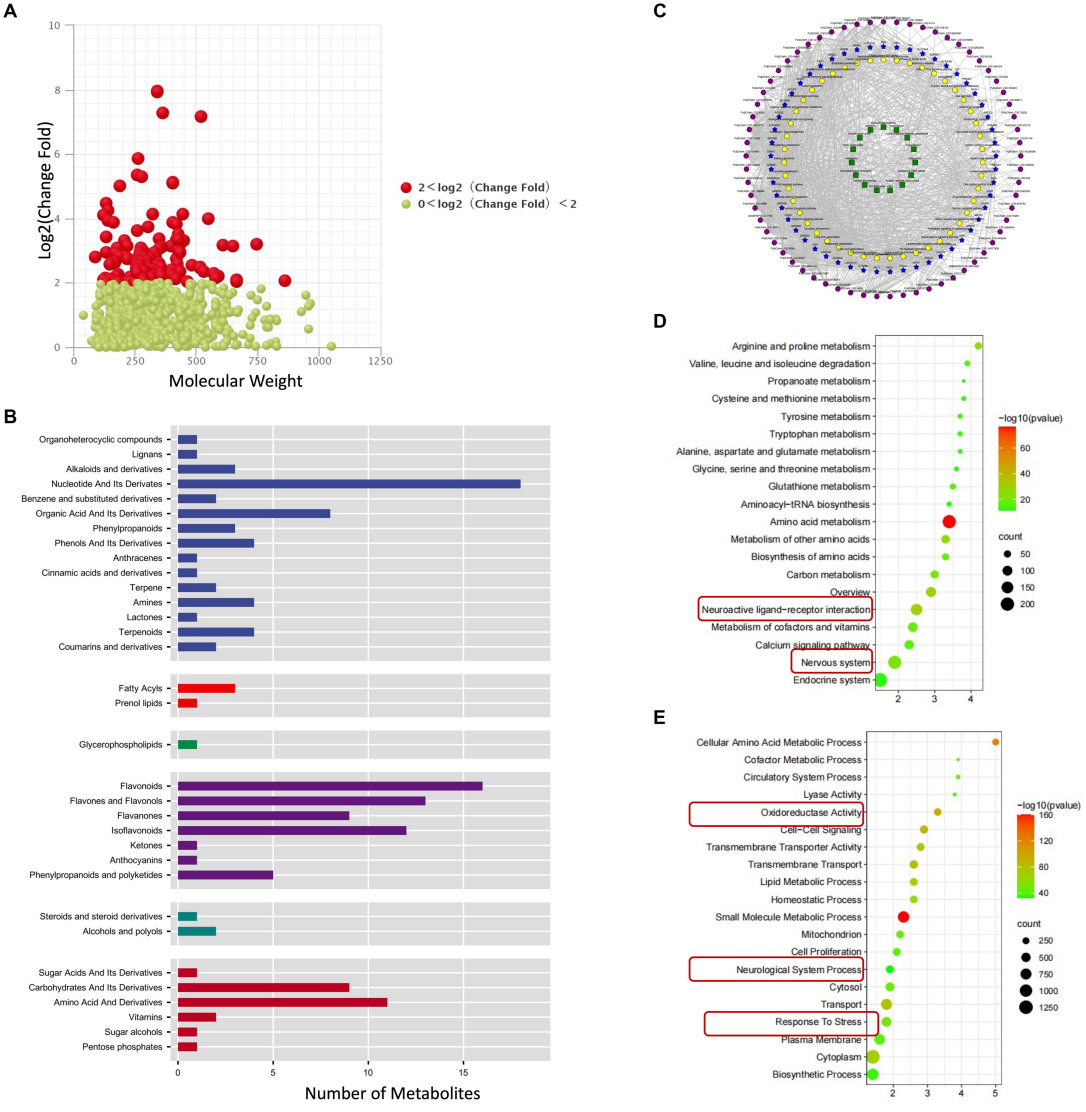
Effect of fermentation on licorice chemical constituents. **(A)** Significantly increased chemicals in the process of fermentation. **(B)** Classification of increased chemicals. **(C)** Bioinformatic prediction of increased chemicals. **(D)** KEGG Enrichment analysis of target genes of highly increased chemicals. **(E)** GO Enrichment analysis of target genes of highly increased chemicals.

In order to further analyze the effect of fermentation on the chemical constituents of licorice, we conducted a differential analysis of the chemical constituents before and after fermentation, and the results showed that 151 compounds were significantly increased (Figure 3A; Supplementary Dataset S2), including probiotic metabolites that are potentially beneficial to the body, involving 9 kinds of carbohydrates and its derivatives, 8 organic acid and its derivatives, including important natural medicinal products, involving 1 anthocyanins, 9 flavanones, 13 flavones and flavonols, 16 flavonoids, 12 isoflavonoids, 4 phenols and its derivatives, 3 phenylpropanoids (Figure 3B). The results suggested that fermented licorice might enhance some medicinal effects of licorice.

### 3.4. In-depth prediction of potential efficacy of fermented licorice via bioinformatics

In order to further predict the potential efficacy of fermented licorice, we used bioinformatics to predict the potential targets of fermented licorice chemical constituents and potential therapeutic diseases. KEGG enrichment analysis showed that the targets of these chemical constituents mainly involved pathways of amino acid metabolism, metabolism of other amino acids, arginine and proline metabolism, carbon metabolism, metabolism of cofactors and vitamins, biosynthesis of amino acids, valine, leucine and isoleucine degradation, calcium signaling and glutathione metabolism (Figure 3C; Supplementary Dataset S3). GO enrichment analysis showed that the targets of these chemical constituents mainly involved small molecule metabolic process, cellular amino acid metabolic process, cell–cell signaling, transmembrane transport, transport, transmembrane transporter activity, cytoplasm, lipid metabolic process and homeostatic process (Figure 3D; Supplementary Dataset S4).

Interestingly, we found that these targets were also related to neuroactive ligand-receptor interaction, nervous system, oxidoreductase activity, neurological system process and response to stress (Figures 3C–E).

**Figure 4:**
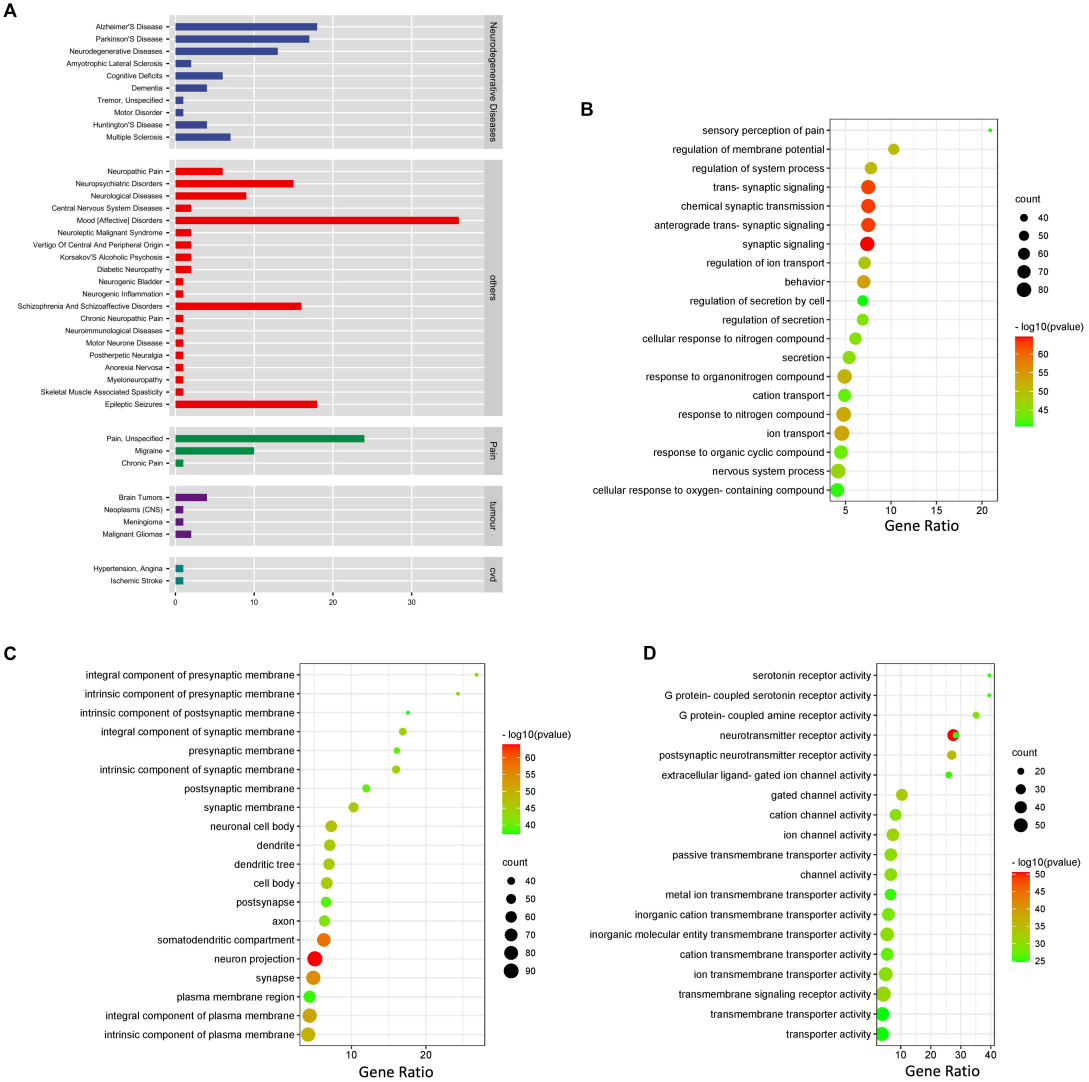
In-depth prediction of potential efficacy of fermented licorice. **(A)** Disease enrichment analysis of target genes. **(B)** Biological process enrichment analysis of target genes associated with neurological diseases. **(C)** Cellular component enrichment analysis of target genes associated with neurological diseases. **(D)** Molecular function enrichment analysis of target genes associated with neurological diseases.

Therefore, we further performed disease enrichment analysis on these targets, and the results showed that these targets were indeed related to neurodegenerative diseases, including Alzheimer’s disease, Parkinson’s disease, Multiple Sclerosis and Amyotrophic Lateral Sclerosis (Figure 4A; Supplementary Dataset S5).

Biological process analysis showed that they mainly involved synaptic signaling, anterograde trans-synaptic signaling, chemical synaptic transmission, trans-synaptic signaling and behavior (Figure 4B; Supplementary Dataset S6). Cellular component analysis showed that they mainly involve neuron projection, somatodendritic compartment, synapse, integral component of plasma membrane and intrinsic component of plasma membrane (Figure 4C; Supplementary Dataset S6). Molecular function analysis showed that they mainly involved neurotransmitter receptor activity, postsynaptic neurotransmitter receptor activity, gated channel activity, ion channel activity and transmembrane signaling receptor activity (Figure 4D; Supplementary Dataset S6).

### 3.5. Verification of potential efficacy of fermented licorice via *in vitro* and *in vivo* experiments

**Figure 5:**
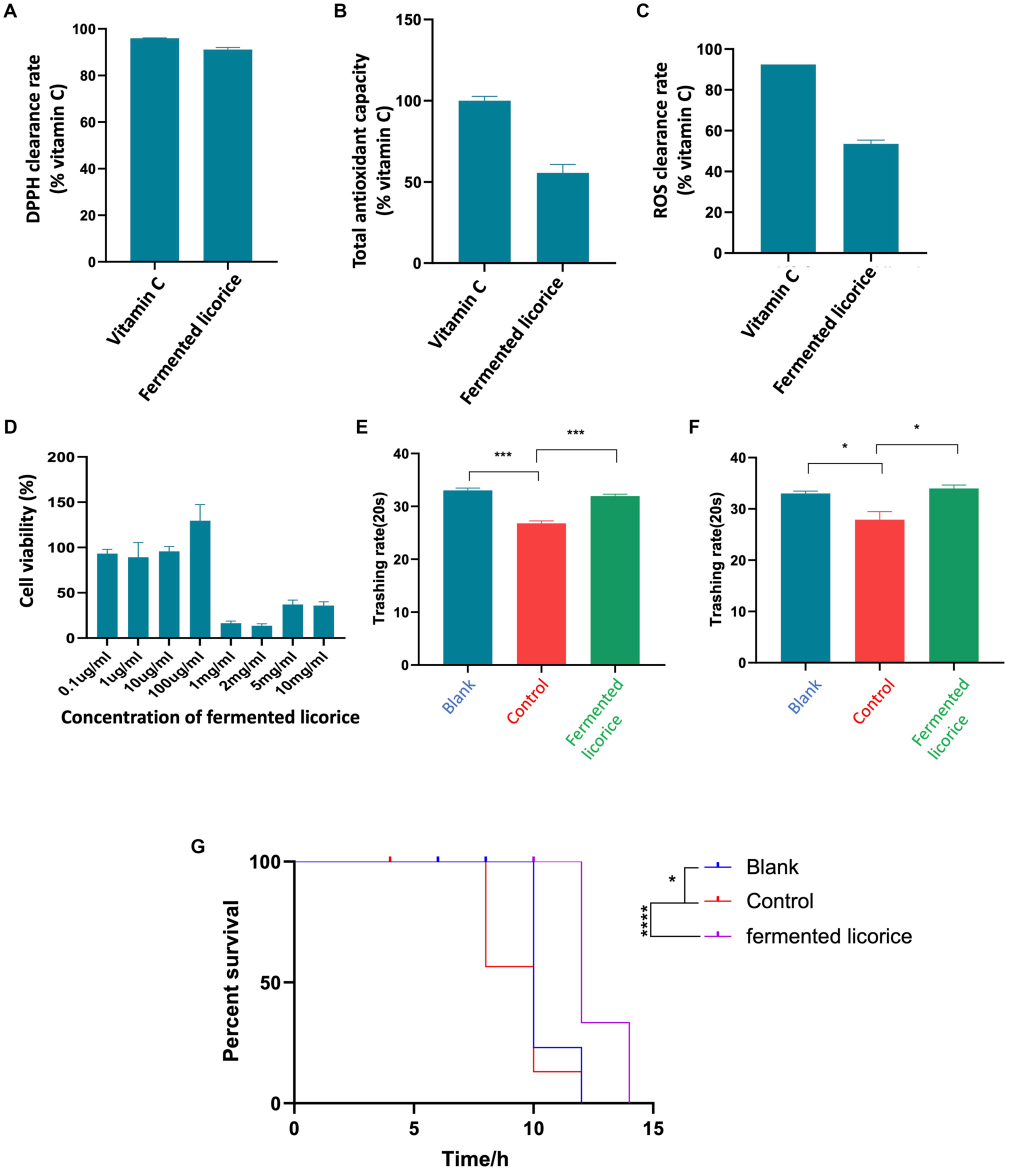
*In vitro* and *in vivo* test of fermented licorice. **(A)** Test of DPPH clearance rate of fermented licorice. **(B)** Test of total antioxidant capacity of fermented licorice. **(C)** Test of ROS clearance rate of fermented licorice. **(D)** SH-SY5Y cell viability under treatment of different concentration of fermented licorice. **(E)** Beneficial effect of fermented licorice on neurodegenerative model of *Caenorhabditis elegans*. **(F)** Beneficial effect of fermented licorice on Alzheimer’s model of *C. elegans*. **(G)** The percent survival of *C. elegans* under heat-stress.

Bioinformatic prediction showed that the targets of fermented licorice chemical constituents involved oxidoreductase activity. To verify this predicted result, we further performed *in vitro* experiment, by using vitamin C as a positive control. The DPPH clearance test showed that fermented licorice had a DPPH clearance ability comparable to that of vitamin C (Figure 5A). The reducing ability test showed that the total antioxidant capacity and ROS clearance rate of fermented licorice could reach 50% of Vitamin C (Figures 5B,C). These results proved that fermented licorice had excellent DPPH scavenging ability and antioxidative ability. Moreover, toxicity testing showed that cell viability could be improved at low concentrations, while at high concentrations there was damage to the cells, which indicated that fermented licorice had excellent safety performance at low concentrations (Figure 5D).

Bioinformatic prediction also showed that the targets of fermented licorice chemical constituents involved neurological system process, response to stress and neurodegenerative diseases. In order to verify the predicted results, we further used the inducible neurodegenerative *C. elegans* model to study the effect of fermented licorice on neurodegenerative diseases. The results showed that fermented licorice at both medium and low concentrations could rescue neurodegenerative-related movement disorders (Figures 5E,F), and could also significantly improve the ability of heat stress (Figure 5G). The results proved that fermented licorice could effectively improve neurodegenerative related movement ability and anti-stress ability.

No studies were focused on the efficacy of probiotic fermented licorice to date. This study firstly discovered and verified that probiotic fermented licorice had excellent DPPH scavenging ability and anti-oxidation ability comparable to that of vitamin C, and had excellent anti-neurodegeneration and anti-stress ability. This discovery would help the development of licorice-related products and promote the development of licorice industry.

## Data availability statement

The datasets presented in this study can be found in online repositories. The names of the repository/repositories and accession number(s) can be found in the article/Supplementary material.

## Author contributions

GX conceived the study. YL participated in design. XW conducted most of the experiments. NK conducted a part of the experiments. GX and YL wrote and revised the manuscript. All authors contributed to the article and approved the submittedversion.

## Funding

This study was supported by National Natural Science Foundation of China (no. 81903738) and the Fundamental Research Funds for the Central Universities (no. 2022-JYB-XJSJJ-031).

## Conflict of interest

The authors declare that the research was conducted in the absence of any commercial or financial relationships that could be construed as a potential conflict of interest.

## Publisher’s note

All claims expressed in this article are solely those of the authors and do not necessarily represent those of their affiliated organizations, or those of the publisher, the editors and the reviewers. Any product that may be evaluated in this article, or claim that may be made by its manufacturer, is not guaranteed or endorsed by the publisher.

## Footnotes

^1^ http://www.genome.jp/kegg/

^2^ http://www.hmdb.ca/

^3^ http://www.lipidmaps.org

^4^ http://bionet.ncpsb.org.cn/batman-tcm/

^5^ https://toppgene.cchmc.org/
